# Different route of hydroxide incorporation and thermal stability of new type of water clathrate: X-ray single crystal and Raman investigation

**DOI:** 10.1038/s41598-017-08152-1

**Published:** 2017-08-22

**Authors:** Mateusz Dulski, Katarzyna M. Marzec, Joachim Kusz, Irina Galuskina, Katarzyna Majzner, Evgeny Galuskin

**Affiliations:** 1 Institute of Material Science, University of Silesia, 75 Pułku Piechoty 1a, 41-500 Chorzow, Poland; 2 Silesian Center for Education and Interdisciplinary Research, 75 Pułku Piechoty 1a, 41-500 Chorzow, Poland; 3 Center for Medical Genomics (OMICRON), Jagiellonian University, Kopernika 7C, 31–034 Krakow, Poland; 4 Jagiellonian Centre for Experimental Therapeutics (JCET), Jagiellonian University, Bobrzynskiego 14, 30-348 Krakow, Poland; 5 Institute of Physics, University of Silesia, Uniwersytecka 4, 40-007 Katowice, Poland; 6 Department of Geochemistry, Mineralogy and Petrography, Faculty of Earth Sciences, University of Silesia, Bedzinska 60, 41-200 Sosnowiec, Poland; 7 Faculty of Chemistry, Jagiellonian University, Ingardena 3, 30-060 Krakow, Poland

## Abstract

Chlormayenite Ca_12_Al_14_O_32_[♦_4_Cl_2_] (♦-vacancy) is partially hydrated micro porouss mineral with hydroxide groups situated at various crystallographic sites. There are few mechanisms describing its hydration. The first one assumes Cl^−^ substitution by OH^−^ at the center of the structural cages (*W-*site). The second one determines the converting a *T*1O_4_ tetrahedron to a *T*1O_3_(OH)_3_ octahedron due to the replacement of oxygen at the *O*2 site by three OH-groups according to the scheme: (^O2^O^2−^ + ^*W*^Cl^−^) → 3 × ^*O*2a^OH. The third mechanism, not considered so far in the case of zeolite-like minerals, includes the hydroxide incorporation in form of hydrogarnet defect due to the arrangement of tetrahedral (OH)_4_ in vacant cages. This yields a strong hydrated phase containing even up to 35% of water more than in any currently known mineral applicable to Portland cement. Moreover, water molecules present in different structural cages are stable up to 355 K while dehydroxylation linked to the gradual loss of only 8% of OH^−^ groups according to 3^*O*2a^OH^−^ → ^O2^O^2−^ +^*W*^OH^−^ + ^g^H_2_O occurs at temperature range from 355 K to 598 K.

## Introduction

The name mayenite was originally given to an isostructural mineral with a composition close to Ca_12_Al_14_O_33_ (C_12_A_7_) discovered at the so-called *type locality*, Bellerberg volcano near Mayen, Eifel, Germany^1^. However, even this mineral name is used nowadays in various scientific disciplines such as chemistry, material sciences, and physics, it is not completely correct in relation to the mineralogy. A recent re-investigation studies of natural mayenite showed that (1) the composition of mayenite from the *type locality* is closer to Ca_12_Al_14_O_32_Cl_2_, and that (2) all samples from other occurrences are either fluorine or chlorine derivatives of Ca_12_Al_14_O_32_[O](C_12_A_7_)^2–5^. Thus, mayenite has been renamed to “chlormayenite” while the name “mayenite” was reserved for a potentially new mineral Ca_12_Al_14_O_33_^4^.

Mayenite compounds can be applied as an ion or electron conductor^6–10^, catalyst in fuel processing^11^, or hydraulic active phase in cement^12–14^ due to their chemical and physical properties such as conductivity, or the potential for water accumulation^15–18^. The mayenite features result from the zeolite-like framework {Al_14_O_32_}^22−^ enclosing six structural cages occupied by two Ca atoms, which leads to an excess of two positive charges^19^. The existence of 33^rd^ extra-framework O^2−^ was postulated as statistically distributed over the six structural cages^19^ and its site is a little bit shifted towards the cage wall^20, 21^. Recent studies of a synthetic fluoride^22, 23^ or chlorine^24^ derivatives of C_12_A_7_ have shown that 2 F^−^ or 2 Cl^−^ ions substitute the extra-framework, O^2−^ occupying the center of the structural cage^25^.

The key issue in order to characterize the synthetic mayenite and their natural counterparts is the analysis of hydration process. It is crucial in the development of new type of cement as well as in solving various geological problems. The process of water incorporation was previously reported due to the scheme: Ca_12_Al_14_O_32_O + ^g^H_2_O = Ca_12_Al_14_O_32_(OH)_2_, where two OH^−^ groups occupying the *W*-site are stable up to 1500 K^26, 27^. However, temperatures associated with dehydration or dehydroxylation processes for these mineral phases have been not determined yet. Another mechanism of hydroxide incorporation is connected with the presence of the “unusual H_2_O molecule” which occupies the empty structural cages according to the scheme: Ca_12_Al_14_O_32_[♦_4_(F,Cl)_2_] + ^g^4H_2_O = Ca_12_Al_14_O_32_[(H_2_O)_4_(F,Cl)_2_]^3, 5^. It has been shown that the molecular water is completely released from the mineral structure at a temperature range between 700–850 K.

This paper presents detailed studies of chlormayenite from Eifel with a simplified formula Ca_12_Al_13.5_Fe^3+^_0.5_O_31.3_(OH)_2.1_[♦_4.7_Cl_1.3_] as well as describes a various route of hydroxide incorporation into its crystal structure. The single crystal X-ray diffraction technique and Raman spectroscopy were applied to develop different models of hydroxylation. Structure stability under various temperatures were analyzed in detail based on *ex-situ* heated grains at 573 K, 773 K and 1073 K as well as *in-situ* one at the temperature range between 293 K and 873 K. Hence, the paper will provide a wide knowledge of the characterization of the mechanisms of hydroxide incorporation, H-bonding scheme and thermal stability of chlormayenite.

## X-ray diffraction and Raman spectroscopy data of natural chlormayenite

**Figure 1:**
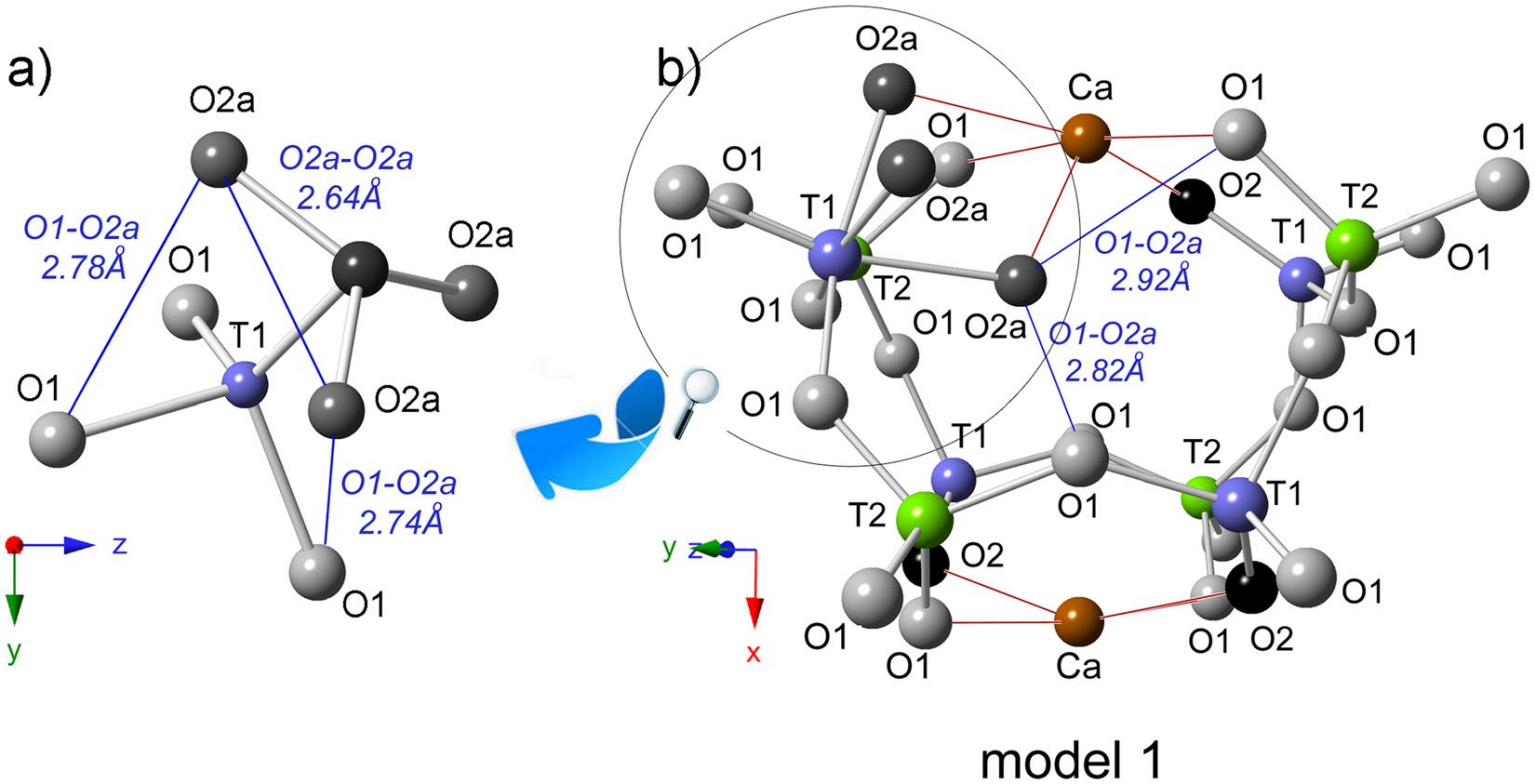
(**a**) Octahedral aluminum coordination at *T*1 and (**b**) potential acceptors for H-bond formation.

**Figure 2:**
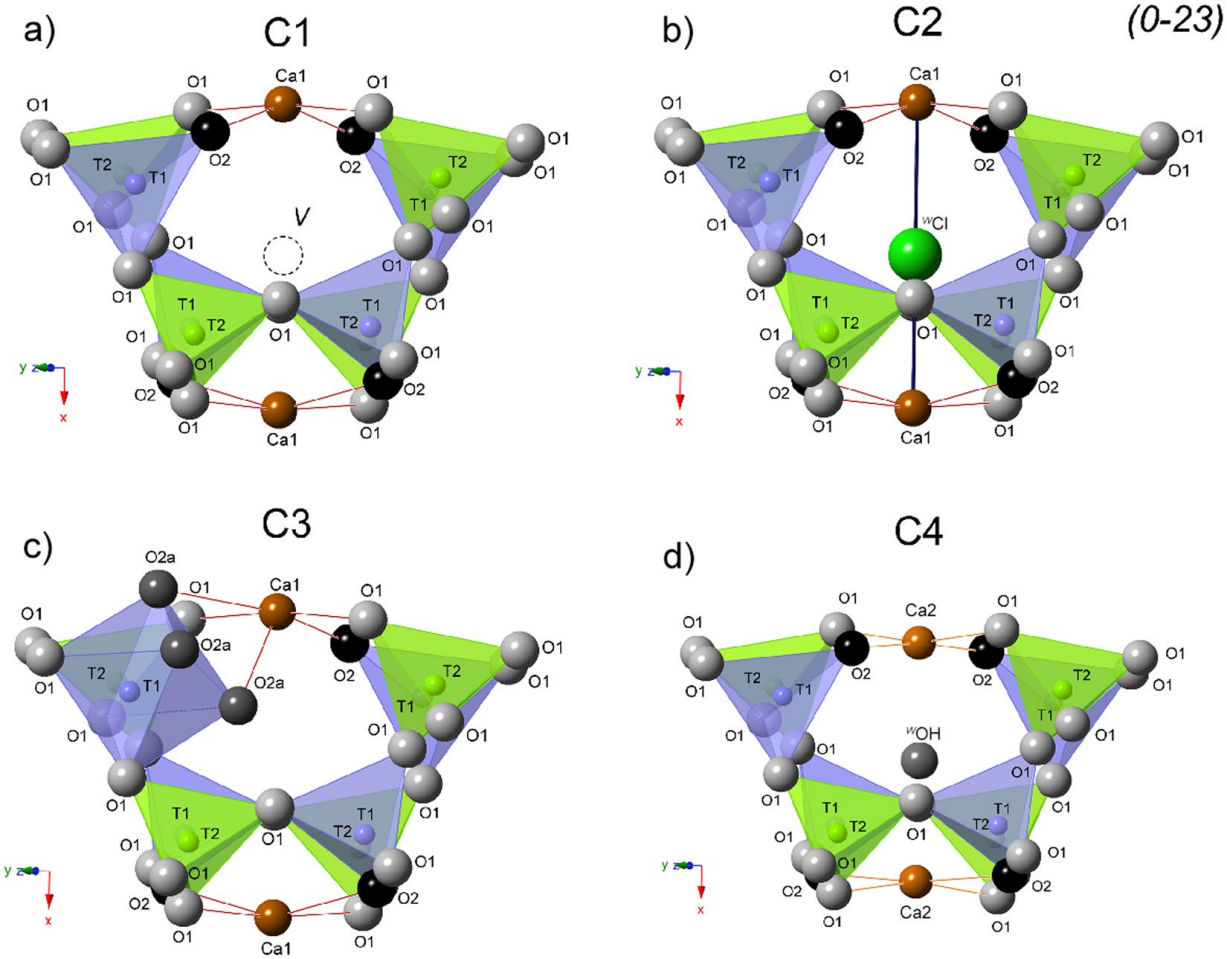
Four different cages of chlormayenite, inclusive extra-framework ions projected onto (0–23). The cage (**a**) C1 is unfilled, (**b**) C2 is filled by ^*W*^Cl^−^ (green sphere), (**c**) C3 in which one of the tetrahedral sites *T*1 (violet) is hydroxylated, whereby ^*O2a*^3OH^−^ replace one oxygen at *O*2 (black spheres). The tetrahedral site T*2* (dark blue) connects four times to *O*1 (dark gray spheres). (**d**) Cage C4 with OH group (gray sphere) situated at the *W*-site which drags the adjacent *Ca*1 (brown spheres) into the cage center (*Ca*2).

The chemical composition of a natural chlormayenite is expressed by ^x1^Ca_12_^*T*1^(Al_7.54_Fe^3+^_0.46_)^*T*2^Al_6_(^*O*1^O_24_^*O*2^O_7.24_♦_0.76_)_32_^*O*2a^(OH)_2.26_^*W*^[♦_4.75_Cl_1.25_] with simplified formula: Ca_12_Al_14_O_31.24_(OH)_2.26_[♦_4.75_Cl_1.25_] and consists of end-members: Ca_12_Al_14_O_32_Cl_2_ (62.5 mol.%) and Ca_12_Al_14_O_30_(OH)_6_ (37.5 mol.%)^2^. X-ray outcomes translate directly to the chlormayenite framework which forms two *T*-aluminum sites {*T*_14_O_32_}. In the Ca_12_Al_14_O_32_Cl_2_ end-member, the *T*2-site is four coordinated with the site symmetry −4, while the *T*1 with site symmetry 3 is situated at the threefold axis. *T*1-aluminum tetrahedra shares only three bonds with *O*1 while the fourth apex is formed by *O*2 oxygen (Fig. 1). The crystal framework is terminated by *O*2 which is not shared with an adjacent tetrahedron. Here, only 1/3 of the cages are occupied by chlorine and this member may be subdivided into 21% of Cl^−^ containing at *W*-site and 42% of vacant *W* cages. The low values of atomic displacement for all tetrahedral framework sites indicates their very strong similarity independent of whether *W*-sites are occupied or vacant (data not shown)^2^. In turns, the Ca_12_Al_14_O_30_(OH)_6_ end-member is characterized by octahedral *T*1 with *O*2 which may partially be replaced by three OH^−^ groups (*O*2a). After such structural change, three of the *O*2a ligands form an equilateral triangle (model 1 in Fig. 1a). Moreover, the short distance of 1.76 Å between *O*2a and *W* requires that the *W*-site must be vacant. All structural cages are filled by a pair of *X* = Ca atoms with site symmetry 2 coordinated by four *O*1 and two *O*2 sites. Two *X*-sites enclose a central cage of about 5 Å in a diameter. Bond lengths for chlormayenite summarized in Table 1. The different cages considered here have following characteristics (1) 4*T*1 and 4*T*2 tetrahedra with vacant *W* in the center (C1); (2) 4*T*1 and 4*T*2 tetrahedra with chlorine in the center (C2); (3) 3*T*1 tetrahedra, *T*1 octahedron, and 4*T*2 tetrahedra with vacant *W*-site (C3); (4) 4*T*1 and 4*T*2 tetrahedra with hydroxide in the center in which the adjacent *X*-sites are shifted towards the center of the cage due to size and charge of the anion (C4) (Fig. 2).

**Table 1:** Selected interatomic distances of untreated chlormayenite^2^ and annealed crystals at 573 K and 773 K and 1073 K.

		100 K [2]	573 K	773 K	1073 K
**Distance [Å]**					
*Ca*1	O1 × 2	2.386 (4)	2.3821 (3)	2.3743 (4)	2.3738 (4)
	O1 × 2	2.496 (4)	2.4924 (5)	2.5027 (11)	2.5042 (15)
	O2 × 2	2.416 (4)	2.4138 (3)	2.4198 (4)	2.4142 (5)
	O2a* × 2	2.256 (4)	2.254 (4)	—	—
	O2a* × 2	2.485 (5)	2.476 (4)	—	—
	^W^Cl	2.854 (2)	2.8508 (4)	2.8508 (12)	2.8386 (17)
*Ca*2	O1 × 2		2.3855 (11)	2.3829 (11)	2.3826 (17)
	O1 × 2		2.786 (11)	2.845 (8)	2.837 (14)
	O2 × 2		2.3584 (12)	2.3600 (7)	2.3564 (11)
	^W^OH		—	2.451 (9)	2.450 (15)
*T*1	O1 × 3	1.790 (4)	1.7864 (3)	1.7842 (4)	1.7790 (5)
	O2*	1.714 (8)	1.7130 (7)	1.7333 (8)	1.7298 (8)
	O2a* × 3	2.098 (5)	2.099 (4)	—	—
*T*2	O1 × 4	1.746 (4)	1.7440 (3)	1.7465 (4)	1.7422 (4)

*Either *O*2 or 3 × *O*2a are occupied.

**Figure 3:**
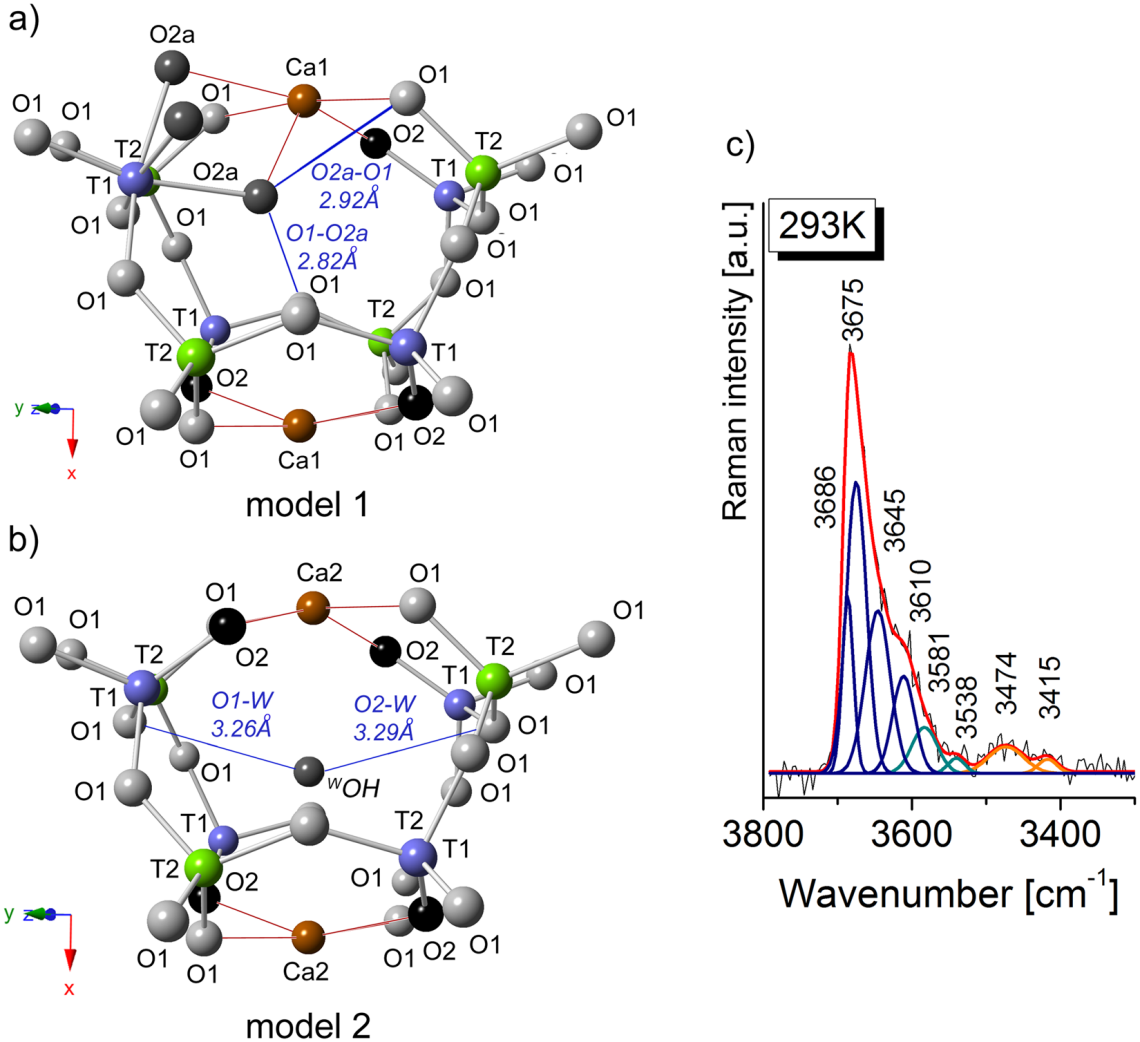
(**a**) Raman spectrum in the hydroxyl stretching region. (**b**,**c**) Two models of hydroxide group distribution observed for the structural cages C3 and C4. The images were projected onto (0–23) in which O*1*, O*2* or O*2a* atoms are potential acceptors for hydrogen bonds.

The potential acceptors of hydrogen bonds in C3 (*O2a* donor) are the adjacent *O*2a of the AlO_3_OH_3_ octahedral with 2.64 Å bond distance (Fig. 1a). The two *O*1 sites defining the triangular face *O*2a-*O*1-*O*1 of the AlO_3_OH_3_ octahedron are the second closest potential H-bond acceptors with a donor-acceptor (D-A) distances of 2.74 Å and 2.78 Å (Fig. 1a). Further potential acceptors are determined for *O*1 of adjacent *T*2O_4_ tetrahedra with D-A distances of 2.82 Å and another *O*1 across the cage with a D-A distance of 2.92 Å (model 1 in Figs 1b and 3a). It turns, X-ray structural refinement of C4 indicated D-A distance between ^*W*^OH donor and O1 or O2 acceptors, respectively as 3.26 Å and 3.29 Å (model 2 in Fig. 3b). Furthermore, an indicator of hydrogen bonds seems also to be the bond valence calculations for the potential acceptor atoms^28, 29^. Here, the calculate valence deficit of around 0.08 vu (valence units) for *O*1 slightly higher than the deficit of the OH-groups at *O*2a (0.02 vu) suggests that none of these oxygen sites are appropriate as an acceptor of a hydrogen bond in case of cage C3^28^. On the other hand, the bond valence increase of ca. 0.23 vu for acceptor oxygen with OH….O distance through ca. 3.20 Å^29^. It suggests that hydroxyl groups at *W*-site like in C4 makes the potential H-bond with acceptors at *O*1 (d_OH…O_ ≈ 3.26 Å) and at *O*2 (d_OH…O_ ≈ 3.29 Å). What is more, the *O*1 acceptor due to shorter d_OH…O_ distance may form a stronger H-bond than oxygen in *O*2 site. However, the real molecular structure may differ from the model structure and bond valence calculations have to be treated with considerable caution. Hence, relying only on such approach, it is difficult to predict real configuration of the hydroxyl group orientation and the most favorable proton arrangement in particular O…O direction.

Therefore, to solve the problem of hydroxide groups in chlormayenite crystal structure the theoretical and experimental Raman studies have been performed. Theoretical approach developed first by Nakamoto and corrected further by Libowitzky has a form of the equation which describes the dependence between the hydroxyl Raman band position and the O…O bond distance (d_O…O_)^30, 31^. Nakamoto diagram predicts an occurrence of Raman bands assigned to the OH modes in the region of 3660–2070 cm^−1^ when O…O distances varied in the range of 2.50–3.20 Å^31^. On the other hand, Libowitzky relation reports that the d_O…O_ = 2.44–3.50 Å are linked to Raman bands in wide wavenumbers range from 3582 to 1758 cm^−1^ ^30^.

The fitting procedures performed for the hydroxyl region on the chlormayenite grains reveals a different type of vibrations which might be separated into three spectral regions of (1) 3750–3580 cm^−1^, (2) 3580–3500 cm^−1^ and (3) 3500–3300 cm^−1^ (Fig. 3c). In the region (1) the bands appear at 3686, 3675, 3645 and 3610 cm^−1^, however, their nature is not quite clear. According to Nakamoto diagram, these bands might correspond to the d_O…O_ distance equal to 3.20, 3.10 and 3.00 Å suggesting the OH group presence within cage C4. However, Libowitzky equation showed, the d_O…O_ distance is not fully conclusive and points to an atypical hydroxyl group arrangements. Moreover, the presence of *O*2a oxygen as a potential proton acceptor should lead to the formation of structural defects, which are statically or dynamically disordered in the mayenite structure. This issue is much more complicated and will be discussed in detail further on. The bands of the region (2) arise probably from the vibration of hydroxyl group occupied *W*-site of cage C4. It is worth to note that Nakamoto diagram does not work here well while Libowitzky formula predicts d_O…O_ bond distance close to 3.10 Å and 3.00 Å. It corresponds to bands with the maximum at 3581 and 3538 cm^−1^ (model 2 in Fig. 3b) and correlates to interpretations for synthetic mayenite^32, 33^. In turns, bands with relatively low intensity observed in the region (3) might originate from an *O*2a-*O*1 bond distance with length equal to 2.92 Å, and 2.82 Å as can be predicted on the base of theoretical XRD data for cage C3.

## X-ray diffraction and Raman spectroscopy data of heat treated chlormayenite crystals

The structural studies and electron microbe analysis (EMPA) were carried out on individual grains and gave the opportunity to obtain their general crystal chemical formulas:

- **573 K**:

^*X*1^Ca_11.4_^*X*2^Ca_0.6_^*T*1^(Al_7.52_Fe^3+^_0.48_)^*T*2^Al_6_(^*O*1^O_24_^*O*2^O_7.36_♦_0.64_)_32_^*O*2a^(OH)_1.92_^*W*^[♦_4.59_Cl*_1.41_], where Cl* ≈ 1.11Cl + 0.3OH with simplified formula: Ca_12_Al_14_O_31.36_(OH)_1.92_[♦_4.59_Cl_1.11_(OH)_0.30_]

- **773 K**:

^*X*1^Ca_10.24_^*X*2^Ca_1.76_^*T*1^(Al_7.52_Fe^3+^_0.48_)^*T*2^Al_6_(^*O*1^O_24_^*O*2^O_8_)_32_[♦_3.93_Cl_1.19_OH)_0.88_] with simplified formula: Ca_12_Al_14_O_32_[♦_3.93_Cl_1.19_(OH)_0.88_]

- **1073 K**:

^*X*1^Ca_10.36_^*X*2^Ca_1.64_^*T1*^(Al_7.52_Fe^3+^_0.48_)^*T*2^Al_6_(^*O*1^O_24_^*O*2^O_8_)_32_[♦_3.88_Cl_1.3_OH)_0.82_] with simplified formula: Ca_12_Al_14_O_32_[♦_3.88_Cl_1.3_(OH)_0.82_]

The small differences in the cation composition with relation to the average composition of ЕМРА: Ca_12_Al_13.5_Fe^3+^_0.5_O_31.3_(OH)_2.1_[♦_4.7_Cl_1.3_] can be explained by the different chlormayenite grains which were taken into consideration through the studies. However, the chemical composition of various grains is slightly different from each other and does not affect the further analysis. Noticeable differences are appearing during the annealing process up to 573 K, 773 K and 1073 K. Annealing at 573 K decreases the number of OH groups at *O*2a from originally 10%^2^ to about 8% wherein the occupancy at the *W*-site slightly increases (Table S1 in supplementary file). Those changes may be described to the scheme: ^*O*2a^3OH^−^ + ♦ → ^*O*2^O^2−^ + ^*W*^OH^−^ + ^g^H_2_O and the data are in agreement with the model. In addition, the refined crystal structure of sample annealed at 573 K points to the splitting of Ca which results in occupancies of 0.973(2) for *Ca*1 and 0.027(2) for *Ca*2. However, the small electron density of OH^−^ at *W*-site is overlapping with the one of Cl^−^ and cannot be refined separately (Table S1 in supplementary file). After crystal annealing at 773 K and 1073 K, OH groups originally located at the *O*2a site are no longer detected (Table S1 in supplementary file) and *T*1 converts to tetrahedral coordination. In addition, the refined structure of crystal annealed at 773 K shows an increase of the electron density at *W*-site and 0.2 Cl^−^ at *W*-site converges to ca. 0.14 additional OH^−^. This central OH attracts adjacent Ca, and leads to split the calcium positions into *Ca*1 and *Ca*2 (due to OH). Since a considerable amount of OH^−^ moving to *W*-site at 773 K, the occupancy factor of OH^−^ has been constrained to the occupancy of displaced *Ca*2 (0.147(6)) while the occupancy of Cl^−^ at *W*-site (0.199(3)) were refined as detached from OH^−^ (Table S1 in supplementary file). The data set of the measurement at 1073 K has been refined identically to the measurement carried out at 773 K. For all refinements, the anisotropic displacement parameters for OH^−^ and Cl^−^ at *W*-site have been set equally (Tables S2-S3 in supplementary file). It is worth to add that the bond distances between *Ca*1-O are practically unaffected by temperature (Table 1) while for the average *Ca*2-O bond distance, only an individual *Ca*2-*O*1 value increases to around 2.84 Å after annealing at 773 K and 1073 K (Table 1). Additionally, similar like for unheated crystal, the presence of hydroxyl groups at *W*-site at all of the annealed samples points to the potential H-bond acceptors at *O*1 (d_OH…O_ ≈ 3.26 Å) and at *O*2 (d_OH…O_ ≈ 3.29 Å). It should as well be noticed that the shift of *Ca*1 to *Ca*2 results in a higher bond valence deficit for the *O*1 (0.230 vu) sites than for *O*2 (0.226 vu) at the OH-populated cage wall. In consequence, the proton-acceptor oxygen in *O*1 site seems to be formed a stronger H-bond than oxygen in *O*2 site.

**Figure 4:**
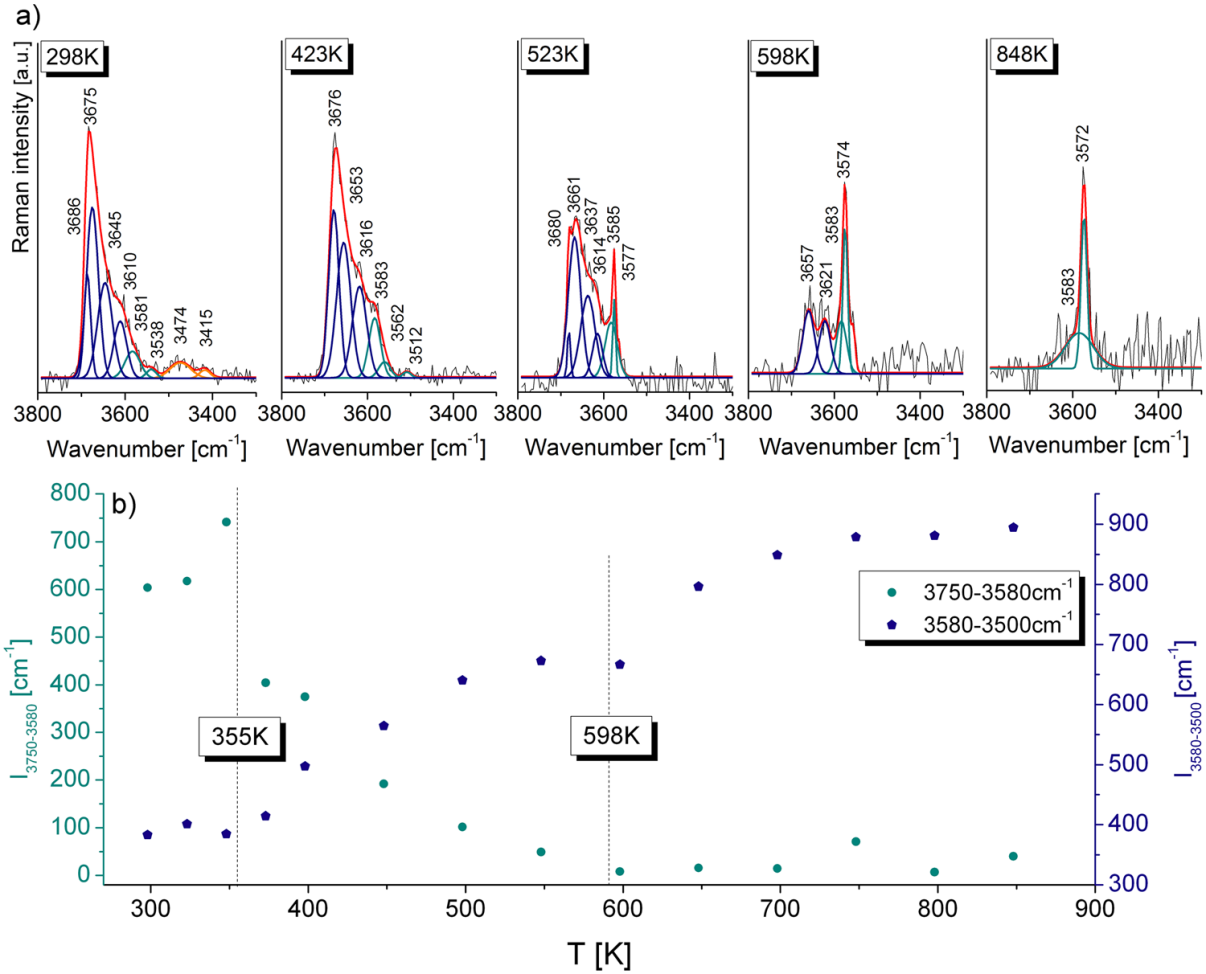
(**a**) The exampled temperature-dependent Raman spectra of chlormayenite in the hydroxyl stretching region. (**b**) The alteration of integral intensity values as a function of temperature in two regions: 3750–3580 cm^−1^ and 3580–3500 cm^−1^.

On the Raman spectrum of unheated crystal, weak bands at 3581 cm^−1^ and 3538 cm^−1^ resulted from the presence of single hydroxyl group occupied the central part of the C4 cage. Further, bands at 3474 and 3415 cm^−1^ reflect a process of partial hydration in which a small number of *T*1 aluminum changes its coordination from tetrahedral to octahedral as a result of further protonated oxygen by the scheme: ^*O*2^O^2-^ + ^*W*^Cl(F)^−^ → ^*O2a*^3OH^−^. Moreover, to determine the dehydroxylation temperature and analyze the thermal stability of ^*O2а*^ОН chlormayenite unit, the temperature-dependent *in-situ* Raman measurements on one single crystal were performed (Fig. 4). The thermal experiment has shown the change of the integral intensity values in two spectral hydroxyl regions which were associated with a reorganization of OH within C3. The intensities of the Raman bands in regions (1) 3750–3580 cm^−1^ and (3) 3500–3300 cm^−1^ decreased while for bands in the region (2) 3580–3500 cm^−1^ increased with rising temperature. Moreover, the disappearance of the band centered at 3686 cm^−1^ suggests vanishing of the absorbed water from a mineral surface while reorganization of OH groups starts above 355 K and ends at about 598 K (Fig. 4a,b).

## Discussion

Recently published papers highlighted the existence of two routes of hydroxide incorporation into the chlormayenite crystal structure. The most energetically favorable mechanisms assumed replacement of one oxygen positioned at the *O*2 site by three OH^−^ groups and converting of a *T*1O_4_ tetrahedron to a *T*1O_3_(OH)_3_ octahedron (model 1 in Figs 1 and 3a) as well as substituting of chlorine atom by one OH^−^ ion into a central *W-*site (model 2 in Fig. 3b). The presence of ^*O2a*^ОН groups distinguishes two O–O distances: *O*2a–*O*1 = 2.92 Å, *O*2a–*O*1 = 2.82 Å which due to Libowitzky correlation ought to be associated with medium strength hydrogen bonds^30^. In turns, the hydroxyl units occupied the *W*-site and give rise to the formation of a weak H-bonds by the location of hydrogen acceptors at *O*1 (d_O…O_ ≈ 3.26 Å) and at *O*2 (d_O…O_ ≈ 3.29 Å) with a bond-valence sum close to 1.73 vu for *O*1 and *O*2 oxygen. The theoretical models are referring to Raman spectrum in the 3600–3200 cm^−1^ range whereas the nature of bands with strong intensity above 3600 cm^−1^ is still not resolved and remains a subject of intense discussion.

**Figure 5:**
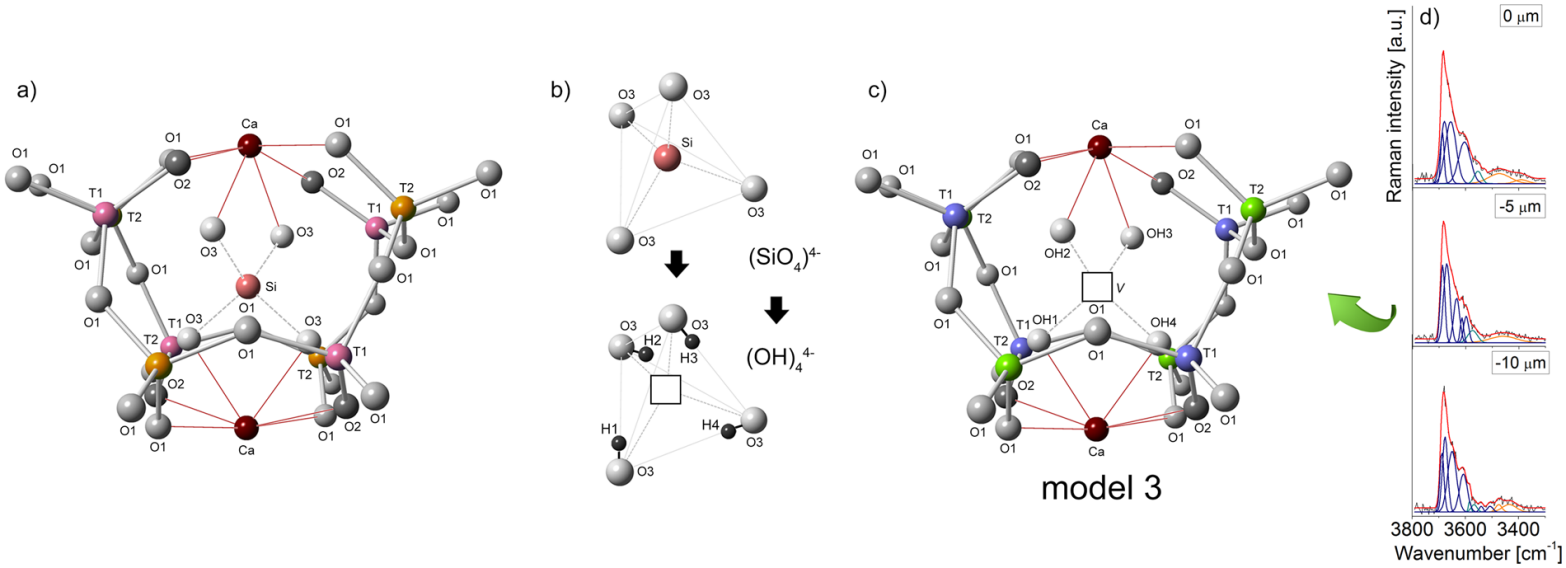
(**a**) Atom arrangement in a grossular-like structure where T*1* and T*2* are ascribed to the position of silicon, (**b**) schema of the hydrogarnet substitution: (OH)_4_ → SiO_4_ where hydrogens occupied an outer position within the tetrahedron between two unshared system edges (modified from ref. 37). (**c**) Atom arrangement assumed in mayenite-like structure with selected hydrogarnet defect where T*1* and T*2* are the silicon position. (**d**) Raman spectra of chlormayenite crystal measured in depth profile and fitted by Voigt function.

In order to solve the origin of undefined chlormayenite bands on Raman spectrum, it is necessary to consider two concepts: (1) the process of hydroxide incorporation into the structure of garnets^34–36^ and (2) electrostatic interaction between a proton and cations (including other protons) due to the formation of hydrogarnet defects (Fig. 5a). Hydrogrossular^37–39^, andradite^40^ and other garnets belong to minerals forming at anomaly low pressures (~ few bars) and temperature of 1000–1350 K while the process of water incorporating into their structure provides formation of hydrogarnet defects in which four O atoms around a silicon vacancy are terminated by hydroxyl groups^34, 35, 38^ (Fig. 5b). A similar condition is crucial to form synthetic Sr-hydrogarnet with a structure closely related to mayenite^36^. However, the degree of hydration strictly depends on the number of OH^−^ ions, the length of shared or unshared polyhedral edges^41^. The high temperature and low pressure conditions are linked to the formation, structurally and chemically similar to garnets, porous mayenite-type phases with the possibility of storing inside the cage, haloids (e.g. chlorine, fluorine), or oxygen according to general pattern Ca_12_Al_14_O_28_O_4_[♦_4_(F,Cl)_2_] (﻿﻿Fig. 5c). In such minerals, the chlorine or fluorine ions distributed in the vacancies, form so-called mayenite defects^42^. Similar conditions of a mayenite-type mineral formation, relative to garnets might favor the emergence of hydrogarnet defects (Fig. 5b and c). However, their appearance will strictly depend on insufficiently high haloid activity, and high abundance of water in the environment. In addition, such groups will not only be linked to the surface but will be distributed in the whole structure (Fig. 5d). Therefore, the water capacity of the mayenite-type structure becomes even a few times higher than previously described in literature^2^.

**Figure 6:**
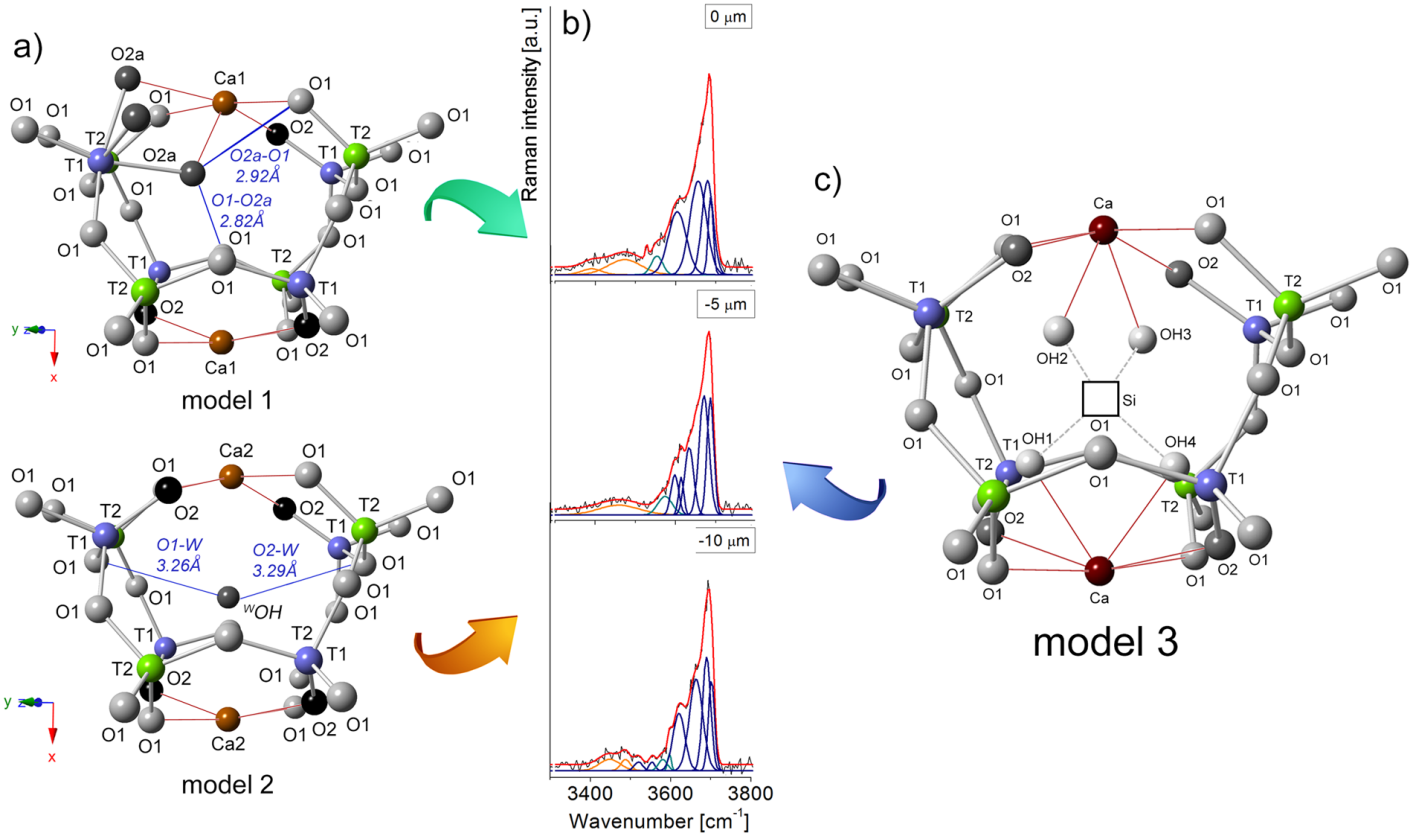
(**a**) Theoretical models of chlormayenite cages based on the diffraction data, (**b**) Raman spectra in hydroxyl stretching region measured in depth profile, (**c**) hydroxyl group arrangement into the structure of chlormayenite. The images were projected onto (0–23) in which O*1*, O*2* or O*2a* atoms are highlighted as potential acceptors for hydrogen bonds.

To shed more light on chlormayenite spectrum in region (1), the obligatory is to look more precisely into the data for garnets. It is due to the correlation between position of hydroxide within polyhedral sites with bands centered between 3700–3500 cm^−1^. Literature reports that the tetrahedral hydrogarnet substitution in hydrogrossular gives rise to overlapping bands centered around 3660 and 3600 cm^−1^ wherein the hydroxide content linearly correlates with the intensity of hydroxyl bands^43^. More detailed studies revealed that band around 3660 cm^−1^ originates from a presence of very weak hydrogen bond scheme within the (OH)_4_ tetrahedron^34^. Here, one can look more closely on the electrostatic interaction between atoms to explain the origin of th﻿e band. The size of this subunit is smaller in relation to typical tetrahedron implying appearance of an electrostatic interaction. As a result, the repulsive force between proton and cations including other protons of OH groups starts to have a stronger influence than attraction force typically considered in a crystalline structure. As a result, such Raman band is shifted towards higher wavenumber. Similar band assignment was also found for a natural and synthetic hydroandradite. However, in the case of these minerals, a shift of hydroxyl bands towards lower wavenumber (3610 and 3560 cm^−1^) might be assigned to the greater average of tetrahedral cation-cation distance within (OH)_4_ group, what implies a decrease of repulsive force and increase the share of attraction ones^44^. Finally, detailed studies for non-cubic garnet such as uvarovite-grossular highlighted that Raman bands observed at 3652, 3640 and 3602 cm^−1^ originate from vacancies on octahedral or dodecahedral cation sites^45^. Thus, the great variability and complexity of the chlormayenite spectrum prove that hydrogarnet substitution probably plays an essential role during water incorporation (model 3 in Fig. 6). Moreover, it may be a key process in clarifying a route of a hydration and help in a description of hydrogen storage mechanism in nominally anhydrous or low hydrous mayenite-type structure minerals. Hence, description of water incorporation will be the first step to expand physicochemical properties of mayenite-type phases, provides unique information about their formation, and finally helps to develop the hypothesis of formation a strongly hydrated garnet-type structure mineral, katoite. This phase is typical for mayenite cement^46^ and gives an opportunity to improve features of building materials, especially sulfur corrosion resistance^47^. It is worth to note here that the hydrogarnet defect leads to the appearance of repulsive force between protons within the (OH)_4_ unit or proton and calcium acting the only way to explain the problem of Raman bands in the 3800 – 3600 cm^−1^ region.

Raman experiment and X-ray diffraction data shed a new light on the temperature stability of ^*O2a*^OH, and (OH)_4_ with relation to ^*W*^OH groups. The temperature-dependent *in-situ* Raman experiment shows that position of bands from regions (1) and (2) are slightly red shifted (Fig. 4a) while the ﻿intensity of bands from region (1) is decreasing with temperature rise. The band near 3682 cm^−1^ disappears at 355 K, supports its assignment to the OH^−^ group vibration of the surface water (Fig. 4b). Moreover, *in-situ* Raman analysis shows that two bands of region (3) arising from OH vibration typically occurs in the octahedral aluminum coordination which disappears at around 355 K as an effect of removal of ^*O2a*^OH moieties. These results confirm a very high instability of AlO_3_(OH)_3_ units and provide proof of the transformation of ﻿structural subunits from octahedral to tetrahedral coordination (Fig. 4). More detailed *ex-situ* XRD and *in-situ* Raman experiments imply that aluminum tetrahedra above 355 K stayed intact without any trace of structural transformation. At the temperature of ~430 K, the most intense bands associated with hydrogarnet substitution are shifted towards higher wavenumber due to hydroxide mobility and weakening of the hydrogen interaction within the (OH)_4_ groups. Similar outcomes were previously reported for hydrogrossular or Sr-hydrogarnet where hydroxyl group was stable up to 573 K^48, 49^ or 673 K^36^. It turned out that a strong diffusivity of hydroxyl moieties might be responsible for a complete disappearance of characteristic chlormayenite bands at temperatures above 598 K. At the same time, temperature-dependent Raman data were correlated with an increase of band intensity from the region (2) according to the dehydroxylation scheme as Ca_12_Al_13.5_Fe^3+^_0.5_O_31.3_(OH)_2.1_[♦_4.7_Cl_1.3_] → Ca_12_Al_13.5_Fe^3+^_0.5_O_32_[♦_4.7_Cl_1.3_(OH)_0.7_] + ^g0.7^H_2_O, where some of ОН groups are moving to the center of the structural cage balancing their charge. According to the literature, the OH group located in a central position formed weak hydrogen bonds which are stable even up to 1500 K^2^.

## Methods

The chemical composition was measured using a CAMECA SX100 electron microprobe operating in wavelength-dispersive spectrometry mode at 15 kV, 10–20 nA and using natural standards (Full description of the methodology is summarized in ref. 2). The single-crystal X-ray data were obtained using SuperNova Dual diffractometer with a mirror monochromator (MoKα, 0.71073 Å) and Atlas CCD detector. The structure was solved by direct methods, with subsequent analyses of difference-Fourier maps, and refined with neutral atom scattering factors using SHELX97^50^. Experimental details for untreated and annealed crystals summarized in supplementary file in Table S3. The Raman experiment was performed using WITec confocal CRM Alpha 300 Raman microscope at excitation laser line λ = 488 nm and CCD detector. The spectra were collected in the range between 4000–120 cm^−1^ with the spectral resolution of 3 cm^−1^ and integration time, 0.3 s for a single spectrum.

## Electronic supplementary material

Supplementary information
