# Supplementary material for: Different route of hydroxide incorporation and thermal stability of new type of water clathrate: X-ray single crystal and Raman investigation
Source: Sci Rep. 2017 Aug 22;7:9046. doi: 10.1038/s41598-017-08152-1 (PMC5567383; doi:10.1038/s41598-017-08152-1)
Supplement: Supplementary file 1 — Supplementary information [file 41598_2017_8152_MOESM1_ESM.docx]

Supplementary information

|  | **573 K** | | | | | | |
| --- | --- | --- | --- | --- | --- | --- | --- |
| *Site* | *Atom* | *x/a* | *y/b* | | *z/c* | *U_eq_* | Occupancy |
| *Ca*1 | Ca | 0.13775(3) | 0 | | 0.25 | 0.00559(4) | 0.973(2) |
| *Ca*2 | Ca | 0.1664(10) | 0 | | 0.25 | 0.00559(4) | 0.027(2) |
| *T*1 | Al | 0.016312(11) | 0.016312(11) | | 0.016312(11) | 0.00461(4) | 0.94 |
| *T*1 | Fe | 0.016312(11) | 0.016312(11) | | 0.016312(11) | 0.00461(4) | 0.06 |
| *T*2 | Al | 0.875 | 0 | | 0.25 | 0.00335(4) | 1 |
| *O*1 | O | 0.03541(3) | 0.44466(3) | | 0.15012(3) | 0.00681(5) | 1 |
| *O*2 | O | 0.18401(3) | 0.18401(3) | | 0.18401(3) | 0.00792(10) | 0.9196(18) |
| *O*2a | O | 0.2005(4) | 0.2847(3) | | 0.1056(3) | 0.0067(7) | 0.0804(18) |
| *W* | Cl | 0.375 | 0 | | 0.25 | 0.0112(2) | 0.2357(18) |
| *W* | OH | - | - | | - | - | - |
|  | **773 K** | | | | | | |
| *Site* | *Atom* | *x/a* | *y/b* | | *z/c* | *U_eq_* | Occupancy |
| *Ca*1 | Ca | 0.13806(10) | 0.0000 | | 0.25 | 0.00517(8) | 0.853(6) |
| *Ca*2 | Ca | 0.1713(8) | 0.0000 | | 0.25 | 0.0080(7) | 0.147(6) |
| *T*1 | Al | 0.017548(13) | 0.017548(13) | | 0.017548(13) | 0.00400(5) | 0.94 |
| *T*1 | Fe | 0.017548(13) | 0.017548(13) | | 0.017548(13) | 0.00400(5) | 0.06 |
| *T*2 | Al | 0.8750 | 0.0000 | | 0.25 | 0.00375(5) | 1 |
| *O*1 | O | 0.03565(3) | 0.44369(4) | | 0.15042(4) | 0.00725(7) | 1 |
| *O*2 | O | 0.18438(4) | 0.18438(4) | | 0.18438(4) | 0.00932(12) | 1 |
| *O*2a | O | - | - | | - | - | - |
| *W* | Cl | 0.375 | 0 | | 0.25 | 0.0115(3) | 0.199(3) |
| *W* | OH | 0.375 | 0 | | 0.25 | 0.0115(4) | 0.147(6) |
|  | **1073 K** | | | | | | |
| *Site* | *Atom* | *x/a* | | *y/b* | *z/c* | *U_eq_* | Occupancy |
| *Ca*1 | Ca | 0.13866(14) | | 0 | 0.25 | 0.00884(10) | 0.863(10) |
| *Ca*2 | Ca | 0.1710(13) | | 0 | 0.25 | 0.0122(10) | 0.137(10) |
| *T*1 | Al | 0.017557(13) | | 0.017557(13) | 0.017557(13) | 0.00597(5) | 0.94 |
| *T*1 | Fe | 0.017557(13) | | 0.017557(13) | 0.017557(13) | 0.00597(5) | 0.06 |
| *T*2 | Al | 0.8750 | | 0 | 0.2500 | 0.00568(5) | 1 |
| *O*1 | O | 0.03570(3) | | 0.44403(4) | 0.15032(4) | 0.01071(7) | 1 |
| *O*2 | O | 0.18441(4) | | 0.18441(4) | 0.18441(4) | 0.01310(13) | 1 |
| *O*2a | O |  | |  | - | - | - |
| *W* | Cl | 0.375 | | 0 | 0.25 | 0.0222(4) | 0.216(5) |
| *W* | OH | 0.375 | | 0 | 0.25 | 0.0222(6) | 0.137(10) |

Table S1. Atomic coordinates and equivalent isotropic displacement coefficients *U_eq_* (Å^2^) of chlormayenite crystals annealed at 573 K, 773 K and 1073 K.

|  | **573 K** | | | | | | | |
| --- | --- | --- | --- | --- | --- | --- | --- | --- |
| *Site* | *U_11_* | *U_22_* | *U_33_* | *U_23_* | | *U_13_* | | *U_12_* |
| *Ca*1 | 0.00590(10) | 0.00659(4) | 0.00428(4) | 0.00057(3) | | 0.000 | | 0.000 |
| *Ca*2 | 0.00590(10) | 0.00659(4) | 0.00428(4) | 0.00057(3) | | 0.000 | | 0.000 |
| *T*1 | 0.00461(4) | 0.00461(4) | 0.00461(4) | 0.00092(3) | | 0.00092(3) | | 0.00092(3) |
| *T*1 | 0.00461(4) | 0.00461(4) | 0.00461(4) | 0.00092(3) | | 0.00092(3) | | 0.00092(3) |
| *T*2 | 0.00305(8) | 0.00350(5) | 0.00350(5) | 0.000 | | 0.000 | | 0.000 |
| *O*1 | 0.00758(10) | 0.00607(10) | 0.00677(11) | 0.00067(8) | | -0.00331(9) | | -0.00154(8) |
| *O*2 | 0.00792(10) | 0.00792(10) | 0.00792(10) | -0.00184(9) | | -0.00184(9) | | -0.00184(9) |
| *O*2a | 0.0079(14) | 0.0051(13) | 0.0072(14) | -0.0016(10) | | 0.0013(10) | | 0.0005(10) |
| *W (Cl)* | 0.0057(3) | 0.0139(3) | 0.0139(3) | 0.000 | | 0.000 | | 0.000 |
|  | **773 K** | | | | | | | |
| *Site* | *U_11_* | *U_22_* | *U_33_* | *U_23_* | *U_13_* | | *U_12_* | |
| *Ca*1 | 0.0053(2) | 0.00653(7) | 0.00368(6) | 0.00108(6) | 0.000 | | 0.000 | |
| *Ca*2 | 0.0130(19) | 0.0058(4) | 0.0054(4) | -0.0002(4) | 0.000 | | 0.000 | |
| *T*1 *(Al)* | 0.00400(5) | 0.00400(5) | 0.00400(5) | 0.00002(4) | 0.00002(4) | | 0.00002(4) | |
| *T*1 *(Fe)* | 0.00400(5) | 0.00400(5) | 0.00400(5) | 0.00002(4) | 0.00002(4) | | 0.00002(4) | |
| *T*2 | 0.00352(10) | 0.00386(7) | 0.00386(7) | 0.000 | 0.000 | | 0.000 | |
| *O*1 | 0.00820(14) | 0.00654(14) | 0.00700(15) | 0.00076(12) | -0.00363(12) | | -0.00184(11) | |
| *O*2 | 0.00932(12) | 0.00932(12) | 0.00932(12) | -0.00202(12) | -0.00202(12) | | -0.00202(12) | |
| *W (OH)* | 0.0059(8) | 0.0143(4) | 0.0143(4) | 0.000 | 0.000 | | 0.000 | |
| *W (Cl)* | 0.0059(4) | 0.0143(5) | 0.0143(5) | 0.000 | 0.000 | | 0.000 | |
|  | **1073 K** | | | | | | | |
| *Site* | *U_11_* | *U_22_* | *U_33_* | *U_23_* | *U_13_* | | *U_12_* | |
| *Ca*1 | 0.0091(3) | 0.01090(8) | 0.00650(7) | 0.00174(7) | 0.000 | | 0.000 | |
| *Ca*2 | 0.020(3) | 0.0098(5) | 0.0071(5) | -0.0009(5) | 0.000 | | 0.000 | |
| *T*1 *(Al)* | 0.00597(5) | 0.00597(5) | 0.00597(5) | -0.00029(4) | -0.00029(4) | | -0.00029(4) | |
| *T*1 *(Fe)* | 0.00597(5) | 0.00597(5) | 0.00597(5) | -0.00029(4) | -0.00029(4) | | -0.00029(4) | |
| *T*2 | 0.00511(11) | 0.00596(8) | 0.00596(8) | 0.000 | 0.000 | | 0.000 | |
| *O*1 | 0.01196(16) | 0.00978(17) | 0.01041(17) | 0.00095(13) | -0.00501(13) | | -0.00265(12) | |
| *O*2 | 0.01310(13) | 0.01310(13) | 0.01310(13) | -0.00309(13) | -0.00309(13) | | -0.00309(13) | |
| *W (OH)* | 0.0108(12) | 0.0279(5) | 0.0279(5) | 0.000 | 0.000 | | 0.000 | |
| *W (Cl)* | 0.0108(5) | 0.0279(6) | 0.0279(6) | 0.000 | 0.000 | | 0.000 | |

Table S2. Anisotropic displacement parameters *U*_ij_ (Å^2^) of chlormayenite crystals annealed at 573 K, 773 K and 1073 K.

|  | *Chlormayenite* | | | |
| --- | --- | --- | --- | --- |
|  | 100 K [1] | 573 K | 873 K | 1073 K |
| Cell parameters | | | |  |
| Space group | $l\overline{4}3d$ | $l\overline{4}3d$ | $l\overline{4}3d$ | $l\overline{4}3d$ |
| Unit cell dimension [Å] | 12.0032(4) | 12.01602(3) | 12.0320(3) | 12.01090(10) |
| Volume [Å^3^] | 1741.86(1) | 1734.930(8) | 1741.86(9) | 1732.71(2) |
| Z | 2 | 2 | 2 | 2 |
| Diffraction data | | | |  |
| Max. Θ-range for data collection | 45.64 | 46.42 | 45.24 | 45.24 |
| Index ranges | -24<h<22  -24<k<24  -22<l<24 | -22≤ h ≤ 24  -23 ≤ k ≤24  -22 ≤ l ≤ 24 | -24≤ h ≤ 15  -18 ≤ k ≤23  -18 ≤ l ≤ 24 | -17 ≤ h ≤ 23  -21 ≤ k ≤20  -15 ≤ l ≤ 23 |
| No. of measured reflections | 59329 | 61338 | 18341 | 36715 |
| Total number of reflections | 1244 | 1293 | 1143 | 1211 |
| No. of observed reflections (I > 2σ (I)) | 1243 | 1282 | 1117 | 1129 |
| The refined structure parameter | | | |  |
| No. of parameters used in refinement | 37 | 39 | 33 | 33 |
| R_int_ | 0.0347 | 0.0316 | 0.0247 | 0.0293 |
| R_σ_ | 0.0074 | 0.0070 | 0.0115 | 0.0157 |
| *R1*, I > 2σ (I) | 0.0117 | 0.0101 | 0.0152 | 0.0154 |
| *R1*, all data | 0.0117 | 0.0104 | 0.0157 | 0.0175 |
| w*R2* (on F^2^) | 0.0346 | 0.0292 | 0.0350 | 0.0323 |
| GooF | 1.169 | 1.229 | 1.120 | 1.046 |
| Δρ_min_ (-eÅ^-3^) | -0.46 close to Ca | -0.41 close to Al2 | -0.76 close to O2a | -0.47 close to Ca1 |
| Δρ_max_ (eÅ^-3^) | 0.78 close to  Ca | 0.21 close to  O2 | 0.20 close to  O1 | 0.23 close to  O1 |

Table S3. Single crystal X-ray diffraction data and structure refinement for chlormayenite measured at room temperature after annealing to 573 K, 773 K and 1073 K compared to the data of untreated chlormayenite^1^.

**Literature**

[1] Galuskin, E. V. et al. A reinvestigation of mayenite from the type locality, the Ettringer

Bellerberg volcano near Mayen, Eifel district, Germany. Mineral. Mag. 76, 707–716 (2012).
